# MoxR effects as an ATPase on anti-stress and pathogenicity of *Riemerella anatipestifer*

**DOI:** 10.1186/s13567-025-01454-7

**Published:** 2025-02-17

**Authors:** Yang Zhang, Yanhao Zhang, Yushan He, Yarong Hou, Xuedi Li, Xueying Yang, Zutao Zhou, Zili Li

**Affiliations:** 1 State Key Laboratory of Agricultural Microbiology, College of Veterinary Medicine, Huazhong Agricultural University, Wuhan, Hubei China; 2 Key Laboratory of Preventive Veterinary Medicine in Hubei Province, Wuhan, Hubei China; 3 Key Laboratory of Development of Veterinary Diagnostic Products, Ministry of Agriculture of the People’s Republic of China, Wuhan, Hubei China; 4 CRISPR and Archaea Biology Research Center, State Key Laboratory of Microbial Technology and Microbial Technology Institute, Shandong University, Qingdao, Shandong China

**Keywords:** MoxR, *Riemerella anatipestifer*, stress, *bat* operon, pathogenicity

## Abstract

Duck infectious serositis is a septicemic disease caused by the bacterium *Riemerella anatipestifer* (*R. anatipestifer*), which affects ducks, geese, turkeys, and other poultry. While outbreaks have been reported worldwide, the exact mechanisms of infection and disease progression remain unclear. Our previous research identified the two-component system PhoPR within the genome of *R. anatipestifer* and demonstrated its association with the bacterium’s pathogenicity. Through multi-omics analysis, we found that PhoP directly regulates the expression of several genes, including *moxR*, within the *Bacteroides aerotolerance* (Bat) operon. However, the function of MoxR in *R. anatipestifer* has not yet been reported. To investigate the impact of MoxR on the expression of the *bat* operon and the pathogenicity of *R. anatipestifer*, we constructed Δ*moxR* and other derivative strains. Our findings revealed that overexpression of MoxR inhibits the transcription of the *bat* operon. Conversely, deletion of *moxR*, along with exposure of *R. anatipestifer* to thermal or oxidative stress, results in increased transcription levels of the *bat* operon. By measuring the survival ability of each strain under stress, we discovered that MoxR is closely associated with the resistance of *R. anatipestifer* to thermal and oxidative stress by influencing the expression of the *bat* operon. Duckling infection experiments, along with adhesion and invasion assays, showed that deletion of *moxR* in *R. anatipestifer* led to decreased pathogenicity, and lower bacterial load in various tissues. Collectively, our findings collectively demonstrate the significant role of MoxR in the anti-stress and pathogenicity of *R. anatipestifer*, providing new insights into its pathogenic mechanisms.

**Supplementary Information:**

The online version contains supplementary material available at 10.1186/s13567-025-01454-7.

## Introduction

*Riemerella anatipestifer* is a Gram-negative, non-spore-forming, encapsulated, short rod-shaped bacillus [1]. It can be easily isolated from infected animals' brains, liver, heart, blood, and other tissues. Under a light microscope, after Wright’s staining, these bacteria can be observed exhibiting bipolar dense staining. This bacterium can cause disease in ducks, geese, turkeys, and other poultry, leading to symptoms such as fibrinous exudation, perihepatitis, meningitis, and splenomegaly observed during necropsy [2]. To date, the disease has been reported in various countries around the world, posing a significant threat to the duck industry [3].

Through RNA-seq and DAP-seq analysis, our laboratory previously identified the Bat operon in the genome of *R. anatipestifer* YM (RA-YM, accession number CP079205.1.) [4]. The two-component system PhoPR regulates the expression of the moxR gene and other genes within this operon.

The Bat operon, initially characterised in *Bacteroides fragilis* (*B. fragilis*) [5], contains the domains DUF58, VWA, and TRP. Its primary function is to provide the bacteria with the necessary reducing power to adapt to oxygen-rich environments during host infections, thereby enhancing their survival and pathogenicity. Research has shown that the Bat operon in *Porphyromonas gingivalis* responds to oxidative stress and improves survival at initial infection sites [6].

Additionally, deleting three genes from the *bat* operon of *Francisella tularensis* increased sensitivity to various stress responses [7]. Studies have also shown that that the proteins encoded by the *bat* operon in *F. tularensis* engage in intricate interactions with other proteins. Notably, the deletion of the *moxR*1 gene results in the inactivity of proteins such as pyruvate dehydrogenase, which leads to decreased resistance to stress [8].

MoxR AAA ATPase is an enzyme involved in various cellular functions, including protein degradation and refolding. It utilises the energy derived from ATP hydrolysis [9]. It possesses Walker A, Walker B, Sensor I, and Sensor II motifs, which are relatively conserved across different species [10]. These motifs play a crucial role in ATP binding and hydrolysis [11], typically forming a functional hexameric ring structure characteristic of AAA family proteins.

This structure of MoxR consists of an N-terminal α/β subdomain and a C-terminal α-helical subdomain, which are connected by a short loop in the middle [12]. The α/β subdomain contains the β hairpin precursor 1 and an inserted helix 2, which is critical for protein interactions [13]. Phylogenetic analysis of MoxR reveals several subfamilies, including MRP, TM0930, RavA, CGN, APE2220, PA2707, and YehL [14].

Research indicates that the MoxR protein may function as a chaperone, with a notable characteristic being its binding to the VWA domain protein [15]. The VWA protein contains a metal ion-dependent adhesion site (MIDAS), which is involved in the incorporation of metal ions, such as Mg^2+^. Additionally, RavA can interact with the inducible lysine decarboxylase LdcI, forming a cage complex that enhances its own ATPase activity and helps it resist acid stress [16, 17].

In *R. anatipestifer*, MoxR is classified within the classic MRP superfamily. Members of this family are typically associated with DUF58, VWA, and TRP proteins to form operon structures, with MoxR positioned at the forefront of the operon. Notably, the Bat operon in *R. anatipestifer* exhibits this structural feature.

This study focuses on MoxR, an ATPase involved in various cellular activities. Our findings reveal that knocking out MoxR reduced the pathogenicity of *R. anatipestifer* in ducklings. qPCR and characterization analysis of derivative strains indicated that *moxR* is significant for stress resistance and proliferation, as it can influence the expression levels of the *bat* operon. These results provide experimental support for understanding the crucial role of MoxR in the pathogenesis of *R. anatipestifer*.

## Materials and methods

### Animals

Ten-day-old healthy Cherry Valley ducks were purchased from the Yongsheng Duck Company (Wuhan, China) and housed in isolated animal rooms maintained at temperatures between 28 and 30 °C. The Research Ethics Committee of Huazhong Agricultural University approved all animal experiments and procedures (approval no. HZAUSW-2018-011).

### Bacterial strains, plasmids, and growth conditions

The bacterial strains and plasmids used in this study are listed in Additional file 1. Additional file 2 lists the primers employed. The RA-YM strain was used as the wild-type (WT) strain, and all other strains were constructed based on this WT. The WT was cultured in Tryptic Soy Broth (Becton, Dickinson and Company, Franklin Lakes, NJ, USA) or on Tryptic Soy Agar (TSA) supplemented with 5% Newborn calf serum (NBS, Newzerum, New Zealand) at 37 °C and 5% CO_2_, using a shaking incubator.

During the screening of gene complement strains, cefoxitin (1 μg/mL, Macklin, China) was added to both TSA and TSB, while nalidixic acid (50 μg/mL, Macklin, China) was incorporated into the TSA. For the screening of other *R. anatipestifer* strains, spectinomycin (100 μg/mL, Macklin, China) must be added to the TSA and TSB, and 50 μg/mL nalidixic to the TSA.

*E.coli* DH5α (Tsingke, China) and *E.coli* BL21 (DE3) were cultured in Luria–Bertani (LB) or on an LB agar plate. Kanamycin (100 μg/mL, Macklin, China) was added according to experimental requirements. Furthermore, when cultivating *E.coli* X7213, diaminopimelic acid (50 μg/mL, Macklin, China) must be added. When creating the recombinant suicide plasmid pRE112-LSR and the recombinant shuttle plasmid pRES-JX, spectinomycin must be added to the culture medium at a final concentration of 100 μg/mL.

### ATP hydrolysis assay

The activity of the His_6_-MoxR protein was assessed using an ATP hydrolysis assay. The reaction system was set up as detailed in Additional files 3 and 4. The configured system was incubated at 37 °C for 1 h, after which the inorganic phosphorus content was measured using the molybdenum blue method. The resulting curves were plotted, with the concentration of His_6_-MoxR or ATP as the x-axis (abscissa) and the absorbance on the y-axis (ordinate).

### Analysis of protein domains and functions

The National Center for Biotechnology Information (NCBI) conducted an analysis of the nucleotide and amino acid sequences of the *KYF39_08995* (*moxR*) gene. They examined its conserved functional sites and utilised InterPro [18] to predict the conserved domains of the MoxR protein. Additionally, cell-Ploc2.0 [19] was employed to predict protein localisation, while BLAST [20] was used to assess the sequence conservation of the Bat operon protein and the nucleotide homology of the genes across different strains of *R. anatipestifer*. Furthermore, MEGA 11 software was utilised to construct an evolutionary tree for the MoxR protein.

### ***Cloning, overexpression, and purification of His***_***6***_***-MoxR and characterisation of enzyme activity***

The recombinant plasmid pET-28a-MoxR, used for the production of His_6_-MoxR, was constructed as follows, using His_6_-MoxR as an example: *moxR* was amplified by PCR using WT genomic DNA as a template and primers 28a-*moxR*-F/R. The resulting DNA fragment was then digested with *Bam*H I and *Sac*I and cloned into the pET-28a vector, which had also been digested with the same restriction enzyme. The plasmid was verified via Sanger sequencing and subsequently transformed into *E. coli* BL21 (DE3).

*E. coli* BL21(DE3) containing pET-28a-MoxR was grown at 37 °C in an LB supplemented with kanamycin until an optical density at 600 nm (OD_600_) reached between 0.4 to 0.6. At this point, 1 mmol/L isopropyl-1-thio-β-d-galactopyranoside (IPTG) was added. After incubating for 21 h at 16 °C, the cells were harvested and resuspended in bacterial lysis buffer (50 mmol/L Tris–HCl, 100 mmol/L NaCl, 1% Triton X-100, 10% glycerin; pH 8.0).

The cells were then crushed three times by a pressure cell disruptor followed by 15-min centrifugation at 10 000 × *g* to keep the supernatant, and the unbroken cells and insoluble fraction were removed. The His_6_-MoxR was isolated from the cell lysates using a Ni–NTA Starose 6 Fast Flow column (Nanotion Biotech., China) that was pre-equilibrated with binding buffer (50 mmol/L Tris–HCl, 100 mmol/L NaCl, 10% glycerin, 5 mmol/L imidazole; pH 8.0). The process involved washing the column with the binding buffer, followed by washing with a buffer containing 50 mmol/L imidazole, and then a gradient elution with 50 to 500 mmol/L imidazole at a gradient of 50 mmol/L. The elution fraction containing His_6_-MoxR was dialyzed in the binding buffer to remove the high concentration of imidazole and concentrated using an ultrafilter (Merck KGaA, Darmstadt, Germany). SDS-PAGE and western blotting with an anti-His-tag antibody were used to confirm the purified protein (ABclonal, China).

His_6_-MoxR protein activity was analysed using ATP hydrolysis assay as mentioned in Materials and Methods. The reaction system was configured as shown in Additional files 3 and 4. After the ATP hydrolysis system was configured, it was incubated at 37 °C for 1 h, and the inorganic phosphorus content was determined by molybdenum blue method, and the curves were plotted with the concentration of His_6_-MoxR or ATP as the abscissa and the absorbance as the ordinate.

### Construction of moxR gene knockout strains, complement strains, knockdown strains, and overexpression strains

The suicide plasmid pRE112 was used to construct the gene deletion strain. The pRE112 was linearised via PCR. The left and right homologous arms of the *moxR* gene were amplified using PCR, with the WT genome as a template. The spectinomycin resistance (*Spc*) gene was amplified using plasmid pIC333 as a template, and the recombinant suicide plasmid pRE112-*moxR*-LSR (which has homologous arms on both sides of the *Spc* gene) was constructed using the homologous recombination method. The pRE112-*moxR*-LSR was transferred into the WT according to the method described by  Zhang et al. [4]. The screen transformants on the TSA plates contained 100 μg/mL spectinomycin and 50 μg/mL nalidixic acid.

The shuttle plasmid pRES-JX was used to construct CΔ*moxR*, WT::*moxRi*, and WT::*moxR*. The pRES-JX plasmid was linearised using PCR, and the WT genome served as a template to amplify the *moxR* gene containing the promoter, the *moxR* antisense sequence and the *moxR* CDS sequence. The plasmid pLMF03 [21] was used as a template to amplify the cefoxitin resistance (*Cfx*) gene. The *moxR* gene and the Cfx gene were used to construct pRES-*moxR*-Cfx using a Gibson assembly with a linear pRES-JX fragment. The *moxR* antisense sequence and CDS sequence were used to construct pRES-JX-*moxRi* and pRES-JX-*moxR* with linear pRES-JX fragments, respectively.

The pRES-*moxR*-Cfx was then transferred into Δ*moxR,* and the transformants were screened on TSA plates containing 1 μg/mL cefoxitin and 50 μg/mL nalidixic acid. The remaining shuttle plasmids were transferred into WT, respectively, and the transformants were selected on TSA plates containing 100 μg/mL spectinomycin and 50 μg/mL nalidixic acid. The constructed pRES-*moxR*-Cfx was linearised using PCR, and the WT genome served as a template to amplify the antisense sequence of *moxR*. The method used to construct WT::*moxRi* was the same as that used to construct CΔ*moxR*::*moxRi*.

Single colonies from each plate were isolated and identified using the primers 16S rRNA-F/R, MoxR-JD-F/R, SPC-JD-F/R, as detailed in Additional file 2, for the identification of Δ*moxR*, CΔ*moxR*, and CΔ*moxR*::*moxRi*. Additionally, the primers JX-ompA-TY-F/R were used for the identification of WT::*moxRi* and WT::*moxR*.

### Total RNA extraction and reverse transcription

After culturing the WT bacteria under normal or stress conditions, the bacteria were collected via centrifugation and the medium was discarded. The total RNA was extracted using the RN01-TRIpure Reagent (Keep, China), and the cDNA was obtained via reverse transcription of the RNA using Hifair® III 1st Strand cDNA Synthesis SuperMix for qPCR (Yeasen, China) according to the manufacturer’s instructions.

### Real-time PCR

Total RNA from WT and its derivatives were extracted and then reverse-transcribed into cDNA, as previously described. The primers MD-QP-F/R were designed (as shown in Additional file 6) to identify internal and external *moxR*. qPCR was conducted in technical duplicates, using 5 μL SYBR qPCR master mix (Servicebio, China), 0.2 μL of each primer (10 μM; listed in Additional file 2), and 4.6 μL diluted cDNA sample in a 96-well PCR plate (Thermo Scientific, Massachusetts, USA). All qPCR reactions were carried out on a CFX96 Connect Real-time System (BIO-RAD, USA) according to the manufacturer’s instructions. The *recA* gene was chosen as the reference gene, and gene expression was quantified using the comparative 2^−ΔΔCT^ method.

### Growth curve analysis

The growth curves of the WT, Δ*moxR*, CΔ*moxR*, WT::*moxRi*, and WT::*moxR* strains were determined. The indicated strains were cultivated until they reached the exponential phase (OD_600_ = 0.6 to 0.8) in TSB. The cultures were harvested by centrifugation and resuspended in TSB to an OD_600_ of 1.0. They were then transferred to fresh TSB medium at a dilution of 1:100. Two hundred microliters of the diluted bacteria in TSB were placed into a 100-well honeycomb plate. The plate was incubated at 37 °C in Bioscreen C MBR (Bioscreen, Finland) for 40 h, with the OD_600_ measured every 30 min throughout the growth period.

### Stress experiment

The survival rates of the WT, Δ*moxR*, CΔ*moxR*, WT::*moxR*, and Δ*batA* strains were assessed under stress conditions. Each strain was transferred to 5 mL of TSB medium at a dilution of 1:100 and cultured to the exponential phase at 37 °C using a shaker set to 200 r/min. After this, the bacteria were collected by centrifugation, and the OD_600_ value was adjusted to 1.0 using TSB. The adjusted cultures were then dispensed into 1.5 mL EP tubes for different treatments as outlined in the experimental protocol.

The oxidative stress group contained 10 mmol/L hydrogen peroxide and was incubated at 37 °C. The heat stress group was incubated at 42 °C, while the control group remained at 37 °C. The treated bacteria were diluted by tenfold multiplicity, and the number of viable bacteria was determined using the pour plate method. Each group included three replicates.

The gene expression levels of WT and WT::*moxR* were assessed under stress conditions. The strains were transferred to a TSB medium and cultured at 37 °C on a shaker at 200 r/min until they reached the exponential phase. Afterwards, the bacteria were collected by centrifugation, and the OD_600_ value was adjusted to 1.0 using TSB. The bacteria were then transferred to a medium containing 5 mL of TSB at a ratio of 1:100.

According to the experimental protocol, the oxidative stress group was incubated with 10 mmol/L hydrogen peroxide at 37 °C and 200 r/min, while the heat stress group was incubated at 42 °C and 200 r/min. The control group was incubated at 37 °C and 200 r/min. Both the treatment and control groups were cultured until they reached the exponential phase. At the end of the culture period, total RNA was extracted from the samples and analysed by qPCR.

### Adhesion and invasion experiments

Duck embryo fibroblasts (DEF) in cell vials were digested using trypsin and then spread into 12-well plates for incubation until the cell density reached 5 × 10^5^ cell/wall. The cells were co-incubated for 2 h at a multiplicity of infection (MOI) of 100 with WT and Δ*moxR* at 37 °C in 5% CO_2_. After this, the cells were washed three times with phosphate-buffered saline (PBS) and digested with trypsin. The resulting cell suspension was continuously diluted and plated onto TSA plates, which were incubated at 37 °C in 5% CO_2_ for 48 h. The number of colonies on the plates was then counted.

Following the colony count, the cells were washed again with PBS, and DMEM F12 containing gentamicin (100 μg/mL, Macklin, China) was added. The medium was incubated for 1 h at 37 °C in 5% CO_2_ to kill any extracellular bacteria, allowing for the quantification of invasive bacteria.

### Pathogenicity analysis

The Δ*moxR* and WT strains were prepared in TSB medium and then centrifuged at 5000 r/min for 3 min. Both strains were resuspended in PBS and centrifuged three additional times. The OD_600_ values of the bacteria were determined. The Δ*moxR* solution was serially diluted to 5.0 × 10^9^, 5.0 × 10^8^, 5.0 × 10^7^, 5.0 × 10^6^, and 5.0 × 10^5^ CFU/mL, and the WT solution was serially diluted to 5.0 × 10^7^, 5.0 × 10^6^, 5.0 × 10^5^ and 5.0 × 10^4^ CFU/mL.

Ten-day-old Cherry Valley ducks were divided into 10 groups, with 10 ducks in each group, as specified in Additional file 7. Each flipper was injected with 0.2 mL of the bacterial solution, while the control group received the same volume of PBS. Observations were made regarding the visible changes in ducklings after the bacterial injections. Deaths were recorded, and the LD_50_ was calculated.

We analysed the survival adaptations of WT and Δ*moxR* strains in ducklings. Fourteen-day-old Cherry Valley ducks were inoculated via flipper injection with 1 × 10^6^ CFU of a 1:1 mixture of WT or Δ*moxR*. The control group receive the same volume as PBS. At 1 day and 2 days post-infection (dpi), heart, brain, lung, spleen, and blood samples were collected from two ducks from each group. The samples were weighed and then homogenised, followed by serial dilutions plated onto TSA plates. We added 100 µg/mL Spc for ΔmoxR to facilitate colony counting.

### Statistical analysis

The LD_50_ of *R. anatipestifer* on ducklings was analysed using IBM SPSS 25 software. Statistical analyses for the experiment were completed using GraphPad Prism version 6.01. Differences between groups were evaluated using Student’s *t-*test. Differences in the growth curves were analysed using two-way ANOVA, while the survival curves were assessed with Log-rank analysis. A *p*-value of < 0.05 was considered to be the threshold for significance.

## Results

### MoxR has an ATPase domain and is conserved in different species

**Figure 1:**
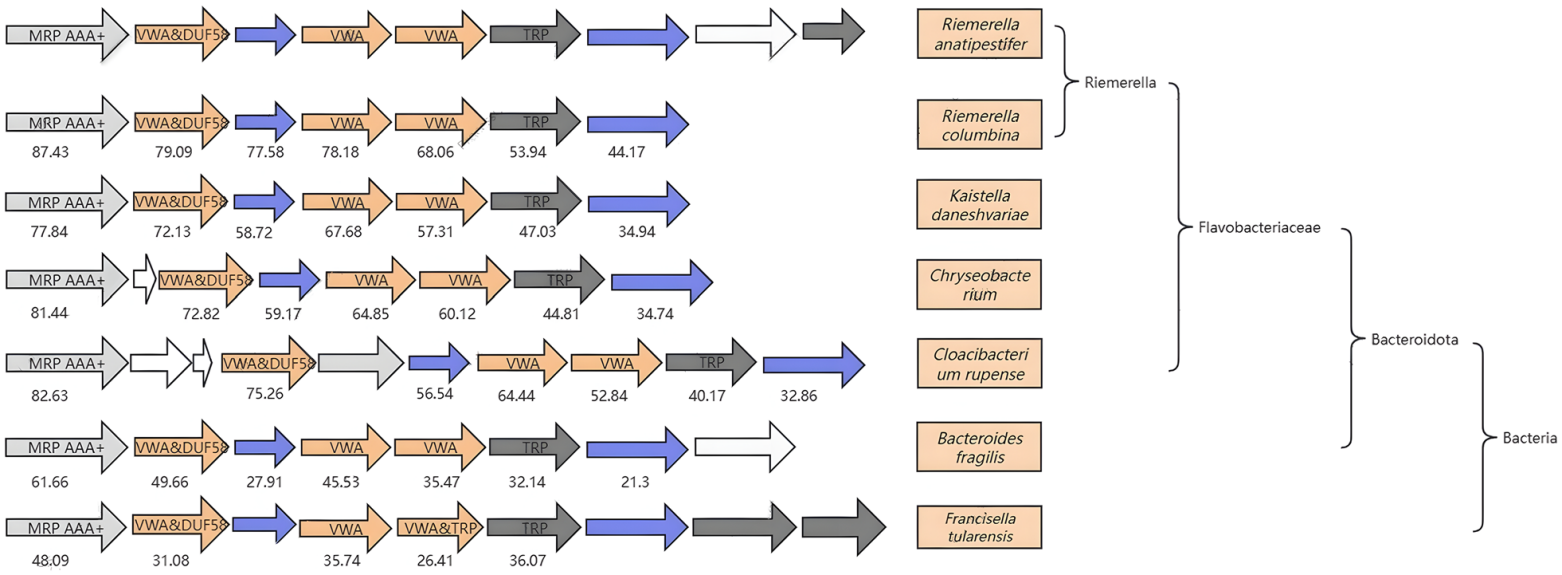
**Amino acid conservation analysis of Bat operon.** The amino acid sequences of Bat operon proteins from different species were compared with those of *R. anatipestifer*. The numbers in the figure show the similarities between the Bat operon proteins of the corresponding strains and the proteins from *R. anatipestifer*. The strains in the same brackets have a closer relationship.

Blast analysis results indicated that the *bat* operon nucleotide sequence is highly conserved among various *R. anatipestifer* isolates, showing over 90% similarity (Additional file 1). The Bat operon amino acid sequence also exhibits significant conservation across different bacterial species. Notably, the MoxR sequence displays the highest level of conservation (Figure 1).

**Figure 2:**
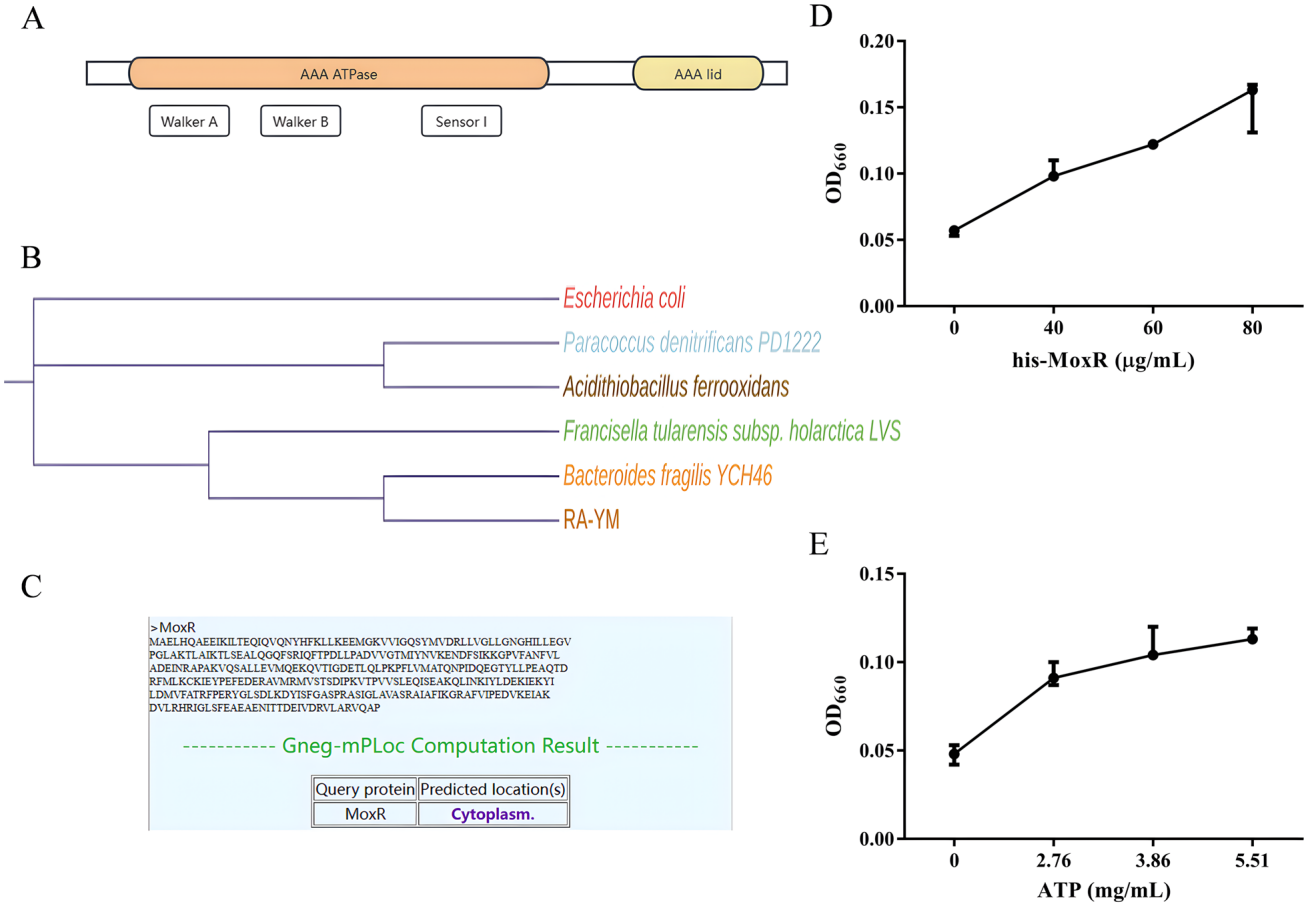
**Functional prediction and validation of MoxR.** **A** The conserved domains of MoxR protein were predicted using the InterPro website. **B** The reported MoxR sequences were queried, and their evolutionary tree was constructed using the MEGA 11 software. **C** The intracellular localization of MoxR was predicted using the Cell-Ploc2.0 website. **D** and **E** The ATP hydrolysis system was configured according to the experimental plan and incubated at 37 °C for 1 h. The content of phosphate groups in the reaction system was determined by the molybdenum blue method to reflect the ATP hydrolysis ability of MoxR. The absorbance of the reaction product at OD_660_ was positively proportional to the content of phosphate groups.

Analyses of conserved domain and protein localisation for MoxR indicate that it contains an ATPase domain at the N-terminus. This domain includes the Walker A, Walker B and Sensor I motifs, which are essential for ATP binding and hydrolysis. The C-terminus features an AAA domain (Figure 2A) whose specific function is not yet known, but it may be involved in interacting with substrate proteins. The evolutionary tree illustrates a close relationship with *B. fragilis* (Figure 2B), and the predicted subcellular localisation of the protein suggests it is a cytoplasmic protein (Figure 2C).

To verify the MoxR protein’s ATPase function, we expressed and purified the His_6_-MoxR fusion protein using *E. coli* BL21 (Additional file 8). Our ATP hydrolysis experiments showed that the concentration of phosphate groups in each reaction system increased with higher amounts of His_6_-MoxR and ATP (Figures 2D and E). This observation indicates that MoxR exhibits ATPase activity.

### Construction and characterisation of *moxR* gene deletion, complement, knockdown and overexpression strains

To investigate the function of MoxR in *R. anatipestifer*, we constructed a *moxR* gene deletion mutant and complement strains, as well as *moxR* knockdown and overexpression strains. We selected colonies resistant to Spc for the deletion, knockdown, and overexpression candidates, while colonies resistant to Cfx were chosen for the complementary strains.

**Figure 3:**
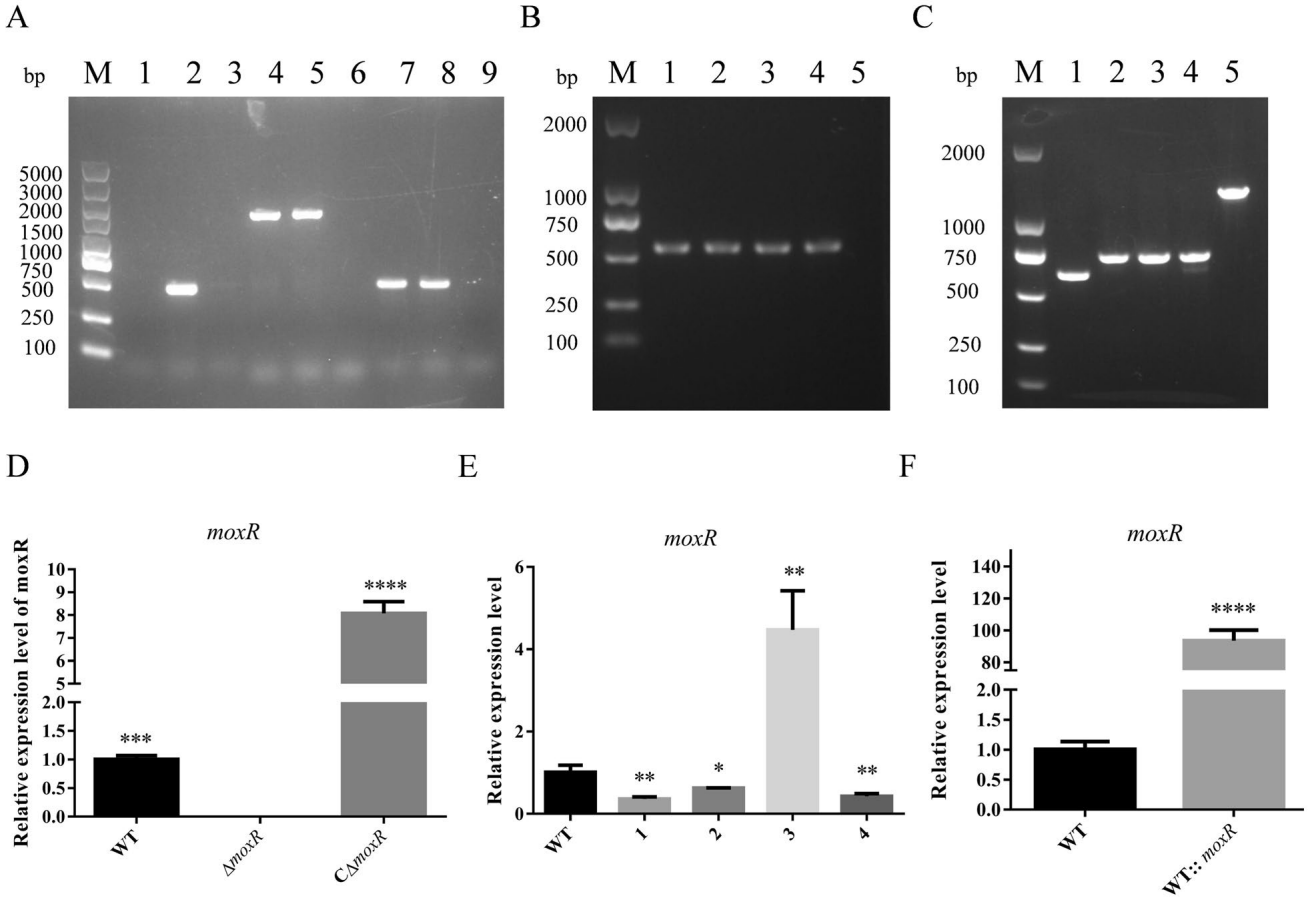
**Identification of constructed strains.** **A** PCR identification results of Δ*moxR*. Lanes 1–3, 4–6, and 7–9 are the identification results of primers MoxR-JD-F/R, 16S rRNA-JD-F/R, and SPC-JD-F/R, respectively. The templates of the three primers are ΔmoxR, pRES-JX-moxR, and ddH_2_O, respectively. **B** PCR identification results of CΔ*moxR*. Primers are MoxR-JD-F/R. Lanes 1–3, 4, and 5 templates are CΔ*moxR*, pRES-JX-moxR, and ddH_2_O, respectively. **C** PCR identification results of WT::*moxR*i and WT::*moxR*. Primers are JX-ompA-TY-F/R. The templates used in lanes 1–4 are WT::*moxR*i of four targets, and the template used in lane 5 is WT::*moxR*. **D** qPCR identification results of Δ*moxR* and CΔ*moxR*. **E** qPCR identification results of WT::*moxR*i with different interference sequence. **F** qPCR identification results of WT::*moxR*. Dates were shown as the means ± SEM. * P < 0.05. ** P < 0.01. *** P < 0.001. **** P < 0.0001.

We then utilised PCR and qPCR methods to identify the candidate strain (Figure 3), employing16S rRNA primers to confirm the strains and sequence them verify their identity as *R. anatipestifer*. The *moxR* gene deletion mutant strain was designated as Δ*moxR*, while the *moxR* gene complement strain was named CΔ*moxR*. The *moxR* gene knockdown strain and the overexpression strain were named WT::*moxRi* and WT::*moxR* respectively. Additionally, the *moxR* gene knockdown strain derived from CΔmoxR was named CΔ*moxR*::*moxRi*.

### MoxR can downregulate the transcription of moxR gene and affect the transcription of *bat* operon

**Figure 4:**
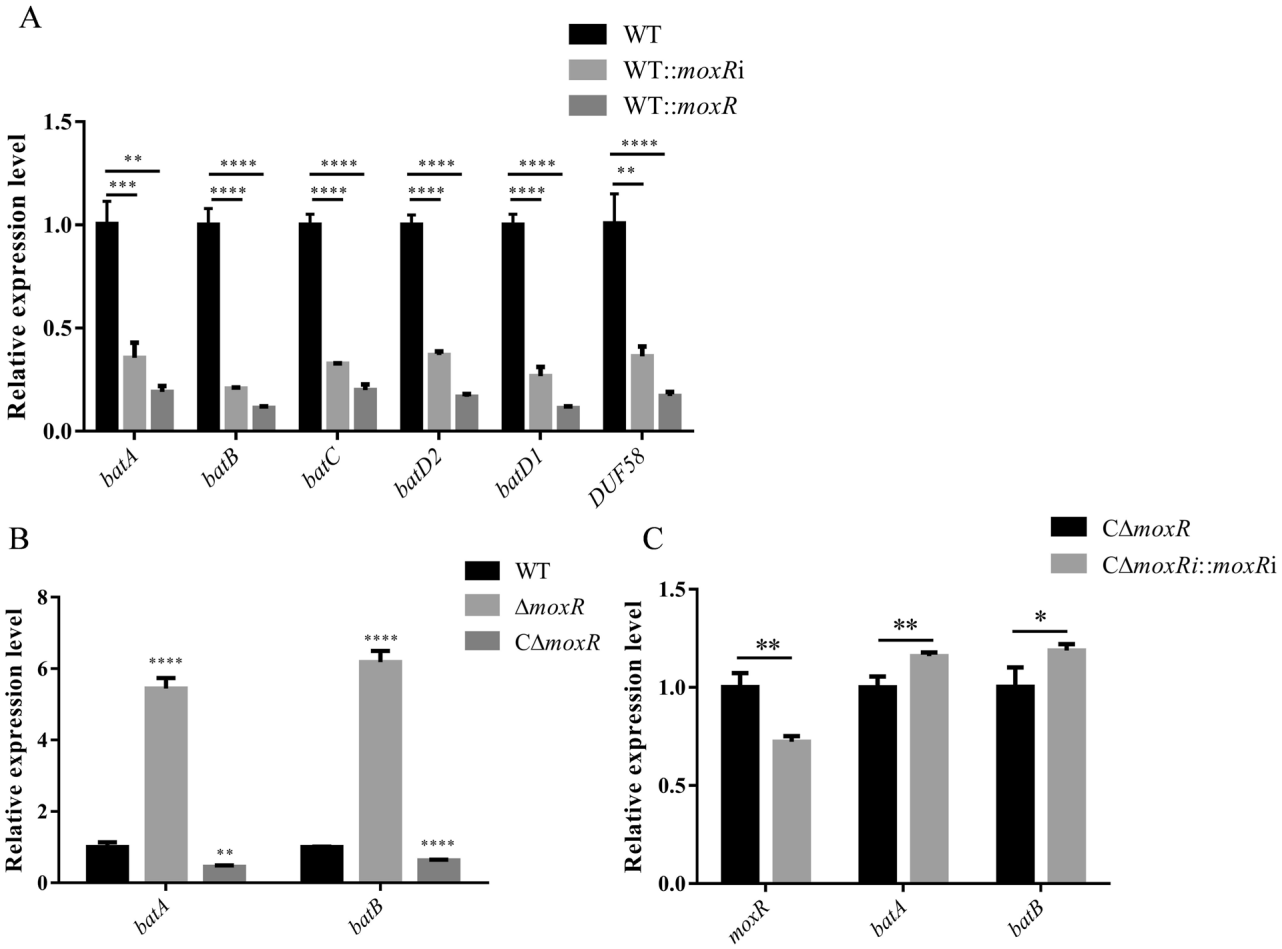
**qPCR analysis of bat operon.** **A** qPCR identification results of WT::*moxRi* and WT::*moxR bat* operon with WT as a control group. **B** qPCR identification results of Δ*moxR*, CΔ*moxR bat* operon with WT as a control group. **C** qPCR identification results of CΔ*moxR*::*moxRi bat* operon with CΔ*moxR* as a control group. Dates were shown as the means ± SEM. *P < 0.05. ** P < 0.01. *** P < 0.001. **** P < 0.0001.

We analysed the effect of MoxR on the *bat* operon using qPCR. The transcription of the *bat* operon increased in the Δ*moxR* strain (Figure 4B). However, when exogenous *moxR* genes were introduced into the WT or Δ*moxR* strains via shuttle plasmid respectively, there was a downregulation of the *bat* operon genes (Figures 4A and B). We utilised the pRES-JX-*moxRi* and pRES-*moxR*-Cfx-*moxRi* shuttle plasmids to introduce antisense RNA (*moxRi*) to downregulate the mRNA level of *moxR* in the WT and CΔ*moxR* strains respectively. The expression changes of other genes in the *bat* operon were then measured.

The downregulation of *bat* operon occurred when pRES-JX-*moxRi* was introduced into the WT strain (Figure 4A). In contrast, the introduction of pRES-*moxR*-Cfx-*moxRi* into CΔ*moxR* resulted in the upregulation of the *bat* operon (Figure 4C). This suggests that seven genes, including *moxR*, displayed consistent mRNA levels and are likely located within the same operon. Additionally, it indicates that the expression of MoxR is involved in the transcriptional regulation of the *bat* operon.

### MoxR is involved in the anti-stress response of *R. anatipestifer*

Pathogenic bacteria encounter various stresses from their host during infection. To investigate the role of MoxR in the anti-stress response of *R. anatipestifer*, we evaluated the survival rates of the WT, Δ*moxR*, CΔ*moxR*, and WT::*moxR* strains in response to heat and oxidative stress.

**Figure 5:**
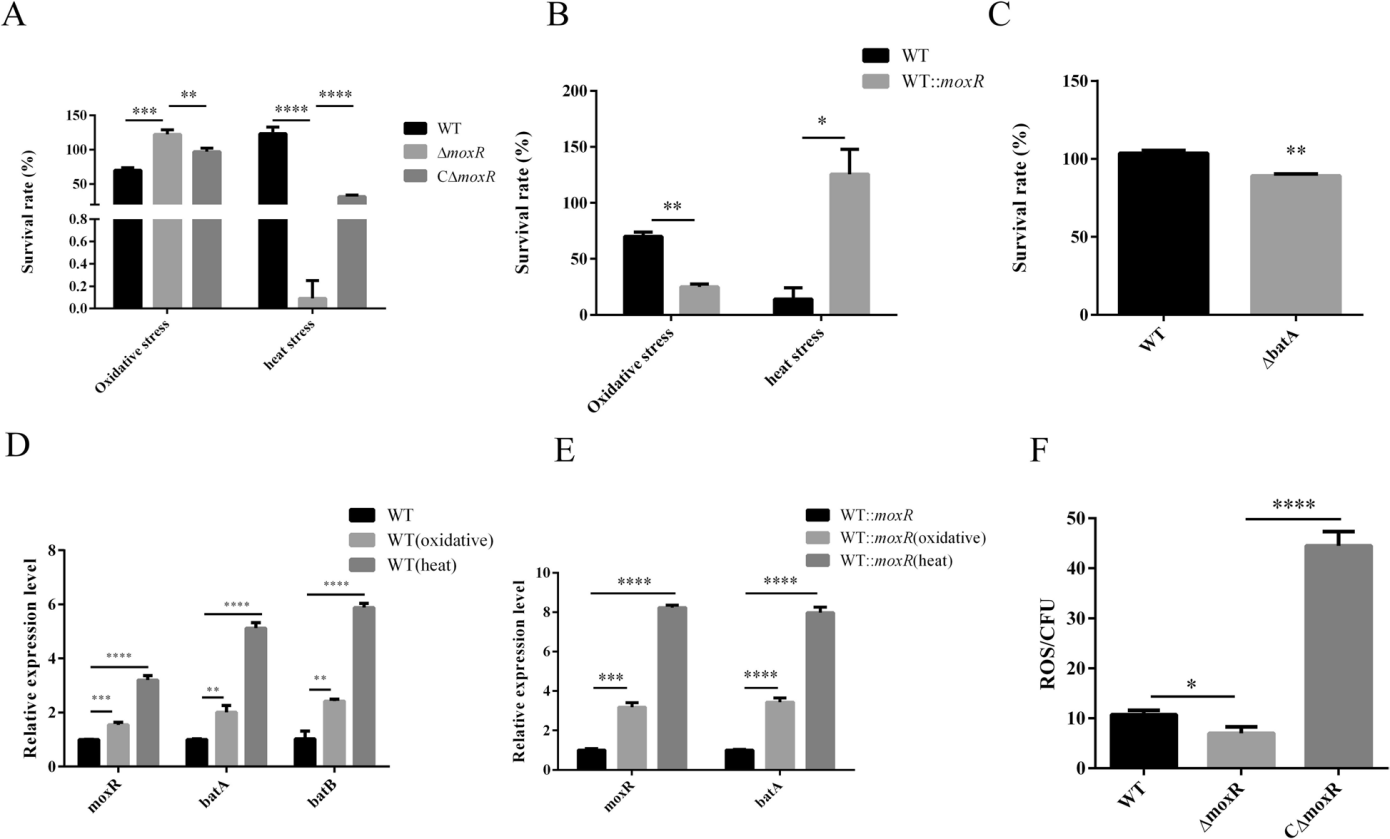
**Determination of anti-stress ability.** **A** and** B** Determination of the ability of Δ*moxR*, CΔ*moxR*, and WT::*moxR* to resist oxidative and heat stress. **C** Determination of the ability of Δ*batA* to resist oxidative stress. **D** and **E** Determination of the effect of stress conditions on the expression of *bat* operon of WT and WT::*moxR*, respectively. **F** Determination of the level of reactive oxygen species in the cells of Δ*moxR* and CΔ*moxR*. Dates were shown as the means ± SEM. * P < 0.05. ** P < 0.01. *** P < 0.001. **** P < 0.0001.

We found that Δ*moxR* exhibited reduced resistance to heat stress compared to the WT, while its resistance to oxidative stress was enhanced. When the *moxR* gene was complemented in Δ*moxR*, its heat stress resistance was restored; however, this led to a significant decrease in resistance to oxidative stress compared to Δ*moxR*. In the WT::*moxR* strain, we observed increased resistance to heat stress compared to the wild strain, but there was a notable reduction in resistance to oxidative stress (Figures 5A and B). To further investigate the impact of *batA* on the ability to resist oxidative stress, we evaluated the survival of Δ*batA* under oxidative stress conditions. Compared to the wild-type strain, Δ*batA* exhibited a reduced capacity to withstand oxidative stress (Figure 5C). Additionally, we measured the in vivo levels of reactive oxygen species (ROS) in the WT, Δ*moxR*, and CΔ*moxR* strains*.* Our findings revealed that the ROS levels of Δ*moxR* were lower than those in the WT, while CΔ*moxR* exhibited higher ROS levels than Δ*moxR* (Figure 5F).

We also cultured WT and WT::*moxR* under stress conditions and found that the expression levels of the *bat* operons in both strains were significantly upregulated (Figures 5D and E). These results suggest that the Bat operon plays a crucial role in the ability of *R. anatipestifer* to resist both internal and external heat stress and oxidative stress.

### MoxR can improve the proliferation ability of *R. anatipestifer*

**Figure 6:**
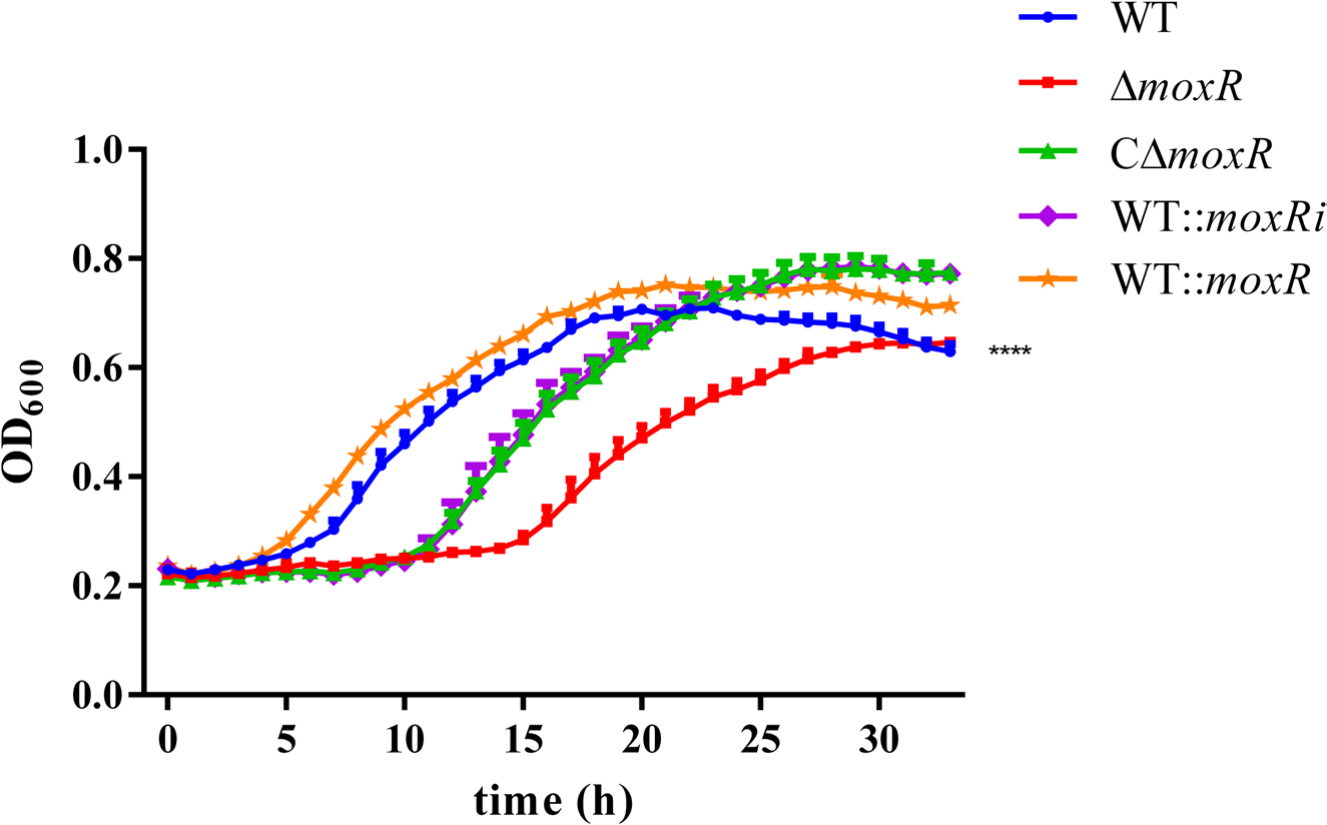
**Growth curves of R. anatipestifer different strains.** The WT, Δ*moxR*, CΔ*moxR*, and WT::*moxRi* and WT::*moxR* were grown to the exponential phase (OD_600_ = 0.6 to 0.8) in TSB. At that point, they were harvested by centrifugation, resuspended to an OD_600_ of 1 in TSB, and then transferred to fresh TSB medium at a dilution of 1:100. OD_600_ was measured every 30 min, and three repetitions were carried out for each strain. The difference in multiplication between WT and Δ*moxR* was compared using the two-factor ANOVA. The results indicated a statistically significant difference between the two groups (*P* < 0.0001). Dates were shown as the means ± SEM. **** P < 0.0001.

To analyse the effect of MoxR on *R. anatipestifer* growth, we measured the growth rate of each strain at 200 r/min at 37 °C, plotting the growth curves based on our findings. The results indicated that the growth rate of Δ*moxR* decreased compared to the WT. However, when complemented with *moxR,* the growth rate of Δ*moxR* increased. Additionally, the growth rate of WT::*moxR* was significantly higher than that of the WT (*P* < 0.0001), while the growth rate of WT::*moxRi* was lower than that of the WT (Figure 6). These findings suggest that MoxR is essential for the growth and proliferation of *R. anatipestifer*.

### MoxR is a virulence-related gene of *R. anatipestifer*

**Figure 7:**
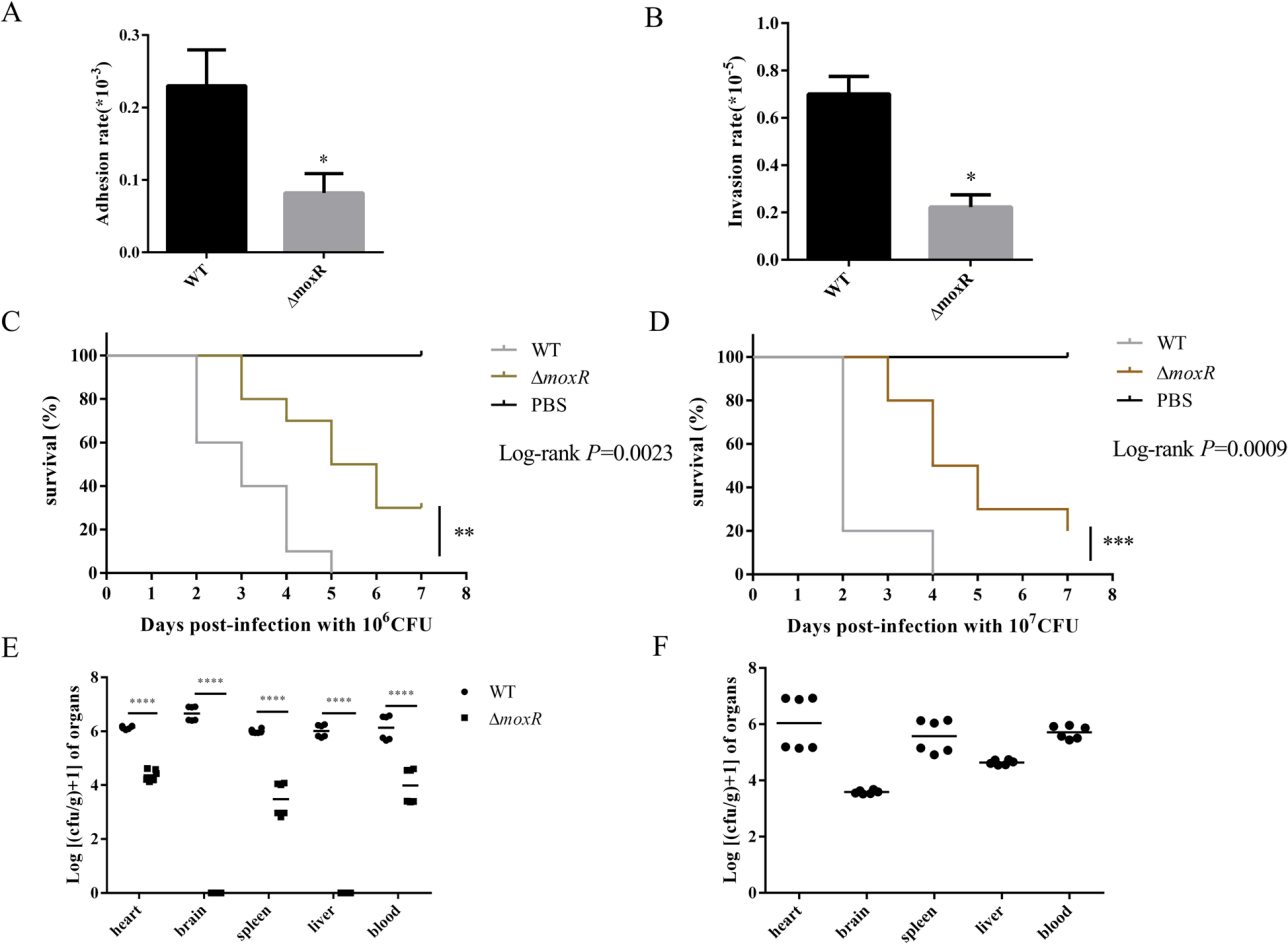
**Pathogenicity analysis of R. anatipestifer different strains.** **A** and **B** DEF cells were incubated with WT and Δ*moxR* (100 MOI) at 37 °C for 2 h, respectively. Then, the cells were digested, and the number of adherent and invasive colonies was counted by the pour plate method. **C** and **D** WT and Δ*moxR* were diluted and inoculated into 10-day-old ducklings through flippers, and the negative control group was inoculated with an equal amount of PBS. The ducklings in each group were observed for 7 days, and the number of deaths was counted. C shows the survival curve at a dose of 10^6^ CFU. D shows the survival curve at a dose of 10^7^ CFU.** E** and **F** WT and Δ*moxR* adjusted to OD_600_ of 1 were mixed 1:1 and diluted in multiple ratios. They were inoculated into 14-day-old ducklings through flippers. The surviving ducklings’ heart, liver, brain, spleen and blood were collected at 24 h and 48 h after inoculation, respectively. After grinding and dilution, they were plated on plates containing Spc resistance and no resistance, and the number of growing colonies was counted. E shows the amount of WT and Δ*moxR* in the tissues 24 h after inoculation. F shows the amount of Δ*moxR* in the tissues 48 h after inoculation. The amount of WT in the tissues exceeds the countable range. Thus, the difference between WT and moxR cannot be analysed. Data were shown as the means ± SEM. * P < 0.05. **** P < 0.0001.

We assessed the adhesion and invasion efficiencies of both the WT and Δ*moxR* strains using duck embryo fibroblast (DEF) cells. Our results indicated that the adhesion and invasion efficiencies of Δ*moxR* to DEF cells were decreased relative to WT (Figures 7A and B). This suggests that MoxR plays a role in the adhesion and invasion processes of *R. anatipestifer*.

The function of MoxR during *R. anatipestifer* infection in the host was further investigated through in vivo adaptation experiments. Pathogenic bacteria were isolated from the hearts, livers, spleens, brains, and blood of ducklings after 24 h and 48 h of infection. The results indicated that the bacterial load of the Δ*moxR* strain was significantly lower than that of the WT in all tested tissues (Figure 7C and D). Only a few Δ*moxR* strains could be isolated from the brain and liver tissues at 24 h, and at 48 h, the Δ*moxR* loads were considerably lower than those found in the other tissues of the brain and liver. Moreover, the WT in all tissues of the ducklings at 48 h was beyond countable levels, allowing only the number of Δ*moxR* colonies to be counted. These findings suggest that MoxR plays a crucial role in the in vivo adaptation and proliferation of *R. anatipestifer*.

The pathogenicity of the WT and the Δ*moxR* strain in ducklings was compared as described in the Materials and Methods section. Ducklings infected with the WT exhibited varying degrees of clinical symptoms after inoculation and succumbed to the infection within 48 h. Specifically, all ducklings in the high-dose group died after 5 days. In contrast, the ducklings infected with the Δ*moxR* strain began showing mortality after 72 h post-infection. Still, the mortality rate in this group was significantly lower than that of the WT group. Notably, more than 20% of the ducklings infected with Δ*moxR* had survived by the end of the observation period (Figure 7E and F).

Statistical analysis determined an LD_50_ of 1.57 × 10^4^ CFU for WT and 5.38 × 10^5^ CFU for the Δ*moxR* strain, indicating that the pathogenicity of Δ*moxR* was significantly reduced. These findings suggest that MoxR plays a crucial role in the infection process of *R. anatipestifer* in the host.

## Discussion

*R. anatipestifer* is considered to be one of the most significant bacterial infections affecting the poultry industry [22]. There are numerous serotypes of *R. anatipestifer*, each exhibiting unique characteristics and lacking cross-protective effects among them [23]. Currently, 21 serotypes of *R. anatipestifer* have been reported internationally [24–27], with a noticeable increase in their prevalence.

In the prevention of duck infectious serositis, vaccines are emerging as a viable alternative to antibiotics. Research has indicated that inactivated vaccines for *R. anatipestifer* provide effective immune protection; however they offer limited cross-protection against different serotypes [28]. Furthermore, the high production costs of inactivated vaccines and their inability to induce a sustained immune response pose significant challenges in effectively combating pathogenic bacteria.

On the other hand, attenuated vaccines can induce a strong immune response in the host and are more cost-effective [29]. Therefore, it is essential to identify suitable vaccine strains and protective antigens that offer cross-protective effects.

It is particularly important to study the virulence factors and pathogenesis of *R. anatipestifer*. The role of MoxR in bacterial resistance to various stresses has been established in other bacteria. In *R. anatipestifer,* MoxR belongs to the MRP superfamily, and the gene coding for the MoxR protein is frequently located at the beginning of an operon. It is often found in the same operon as proteins containing the VWA and TPR domains.

Additionally, VWA domain proteins typically help localise MoxR to substrate proteins [15, 30]. We conducted a homology analysis of the Bat operon in *R. anatipestifer* and found the sequence homology varies based on the evolutionary relationships among different species. Our evolutionary tree analysis of MoxR revealed that *R. anatipestifer* is closely related to *B. fragilis*, followed by *Francisella tularensis* (*F. tularensis*), consistent with their classification. Based on these findings, we speculate that the MoxR proteins in these species may exhibit functional consistency.

The transcription levels of the *bat* operon genes in Δ*moxR*, CΔ*moxR*, and WT::*moxR* were measured. The deletion of *moxR* in *R. anatipestifer* resulted in a significant increase in the transcription of the *bat* operon. Conversely, repression of *moxR* mediated by iRNA led to decreased transcription levels. We interpret this phenomenon to mean that the deletion of *moxR* relieves the transcriptional repression of the downstream Bat operon by MoxR. In contrast, iRNA-mediated interference with mRNA affects the transcription and translation of the entire *bat* operon.

To further validate our hypothesis, we overexpressed *moxR* in the WT using a shuttle plasmid. This resulted in a decrease in the transcription level of the endogenous *moxR*, which was accompanied by the inevitable downregulation in the transcription level of the *bat* operon. MoxR may exhibit negative feedback regulation on its transcripts, and this effect continues even when MoxR is expressed complementarily (Figure 5B). This negative feedback regulation ensures the efficient execution of normal cellular activities without wasting resources [31]. A similar mechanism can be observed in the RepABC operon, which encodes the ATPase RepA involved in plasmid replication and distribution. In this case, RepA negatively regulates the expression of the operon [32].

To further analyse the inhibitory effect of MoxR on *bat* operons, we conducted qPCR analysis on the *bat* operon in both WT::*moxRi* and CΔ*moxR*::*moxRi.* Our findings indicated that when *moxR* is present in the genome, changes in *moxR* transcription levels affect the transcription of the downstream *bat* operon, which aligns with observations in WT::*moxR* and other strains. Conversely, when *moxR* is overexpressed from a shuttle plasmid, the downregulation of its expression does not lead to a corresponding downregulation of the *bat* operon; instead, it actually upregulates their expression. This upregulation may be attributed to reduced repression of the *bat* operon by MoxR. Overall, the expression levels of all genes were consistent, suggesting that they may be located within the same operon sequence, and the knockdown of MoxR may lead to increased expression of *bat* operon.

During *R. anatipestifer* infection, the bacterium faces various stressors, including heat, oxidative, and acid stress [33]. The importance of MoxR in stress resistance and its chaperone function has also been demonstrated in other bacterial species [7, 34]. For instance, Bacteroides with an insertional deletion of the *batD* showed reduced resistance to oxygen [35], which also impacted their growth in different media [5]. The OxyR gene is essential for the resistance of *B. fragilis* to oxidative stress; its deletion significantly lowers bacterial survival rates in H_2_O_2_ [36].

To investigate the role of MoxR in the stress resistance of *R. anatipestifer*, we assessed the survival of WT, Δ*moxR*, CΔ*moxR* and WT::*moxR* under heat stress and oxidative stress conditions. We found that the expression level of *moxR* was closely correlated with the survival of *R. anatipestifer* under heat-stress conditions. In contrast, the expression level of genes such as *batA* was more closely associated with the survival of *R. anatipestifer* during oxidative stress. We evaluated the expression levels of the *bat* operon in both the WT and WT::*moxR* strain under stress conditions. Our observations indicated a synchronous upregulation of mRNA levels for both *moxR* and *batA* under heat and oxidative stress compared to the normal treatment group.

We hypothesise that stress conditions lead to the deregulation of MoxR’s inhibitory effect on its own gene. This results in increased expression of *moxR* and the subsequent upregulation of downstream gene expression (Figure 5E), ultimately providing the pathogen with the resources needed to withstand stress. In our analysis of the growth characteristics of *R. anatipestifer*, we observed that MoxR influences bacterial proliferation. When *moxR* was complemented in Δ*moxR*, its growth rate did not fully restore compared to the WT. This may be attributed to the downregulation of the expression of the *bat* operon, which increases the level of oxidation in the bacterium, thereby affecting the growth rate of *R. anatipestifer*.

Previous studies have reported that the MoxR, VWA1, and TPR1 proteins encoded by the *moxR* operon in *F. tularensis* LVS can interact with pyruvate dehydrogenase and oxoglutarate dehydrogenase [8], playing significant roles in essential cellular activities. Additionally, mutations in bacterial redox-related genes can lead to increased sensitivity to the external environment, as reducing power is crucial for the survival and functionality of bacteria. High oxidation environments can deactivate specific enzymes within bacteria and directly damage them [5]. However, prolonged exposure to such conditions triggers a variety of metabolic responses in bacteria to generate significant reducing power [36].

Deleting *moxR* may lead to the dysfunction of certain proteins [8], which can disrupt essential bacterial activities. Moreover, downregulating the *batA* gene can accumulate reactive oxygen species, negatively impacting bacterial performance. Consequently, deleting moxR or downregulating genes like *batA* will reduce the growth rate of *R. anatipestifer*.

Experiments on duckling infections, along with adhesion and invasion assays, have demonstrated that MoxR plays a crucial role in the pathogenicity of *R. anatipestifer* in ducklings. The mortality and death rates observed in the Δ*moxR* strain post-inoculation were reduced compared to the WT strain. This indicates that deleting MoxR impairs the bacterium’s ability to proliferate and adapt to the host’s survival conditions.

Furthermore, the absence of MoxR results in a weakened ability of *R. anatipestifer* to regulate the expression of the *bat* operon, leading to reduced resistance to stress within the host. This impairment affects the bacterium’s adaptation to and colonisation of host cells and tissues. Additionally, MoxR may function as a potential molecular chaperone [37–40], and the loss of specific protein functions due to its deletion could help the Δ*moxR* strain to adapt to the host environment.

Overall, the reduction in pathogenicity of the Δ*moxR* strain in ducklings can be primarily attributed to the combined effects of these factors.

In conclusion, our study clearly demonstrates the significant role of MoxR in the growth, stress resistance, and pathogenicity of *R. anatipestifer.* This conclusion is based on comprehensive in vivo and in vitro experiments and an analysis of its regulatory role in gene expression. The conservation of *moxR* across different serotypes of *R. anatipestifer* and its involvement in pathogenicity highlights its potential as a promising candidate gene for developing attenuated vaccines aimed at multiple serotypes of *R. anatipestifer*.

## Supplementary Information

**Additional file 1. Strains and plasmids involved in this study**. The plasmids and strains involved in this study are listed in the table.

**Additional file 2. Primers used in this study**. Primers for PCR and qPCR experiments were used in this study.

**Additional file 3. ATP hydrolysis system with different concentrations of MoxR**. The content of ATP was controlled to remain unchanged, and the ATP hydrolysis system was configured. After incubation, the content of phosphate groups in each system was determined by the molybdenum blue method.

**Additional file 4. ATP hydrolysis system with different concentrations of ATP. **The content of His_6_-MoxR was controlled to remain unchanged, and the ATP hydrolysis system was configured. After incubation, the content of phosphate groups in each system was determined by the molybdenum blue method.

**Additional file 5. Nucleotide conserved analysis of** ***bat*** **operon.** The conservation of the nucleotide sequence of the *bat* operon of the RA-YM strain in *R. anatipestifer* was analysed by blast. The comparison results showed that the sequence conservation of the 48 strains of RA was above 93%, and the sequence of the same colour indicates that their conservation is above 99%.

**Additional file 6. Schematic diagram of endogenous** ***moxR*** ***gene identification.*** Primers MD-QP-F\R are used to distinguish *moxR* genes in the genome or plasmids. The primer MD-QP-F is complementary to the coding sequence of moxR, and the primer MD-QP-R is to the coding sequence of DUF58, the downstream gene in the genome. As a result, only the mRNA transcribed from the endogenous *moxR* can be detected.

**Additional file 7. Vaccination schedule**. The dose inoculated in each group in the lethal dose of RA to ducklings.

**Additional file 8. Purification and identification of His**_***6***_**-MoxR.** His_6_-MoxR protein was expressed and purified by prokaryotic expression system, and SDS-PAGE and western blot identified the purified protein.

## Data Availability

The datasets used and analysed during the current study are available from the corresponding author upon reasonable request.
