# Supplementary material for: MoxR effects as an ATPase on anti-stress and pathogenicity of Riemerella anatipestifer
Source: Vet Res. 2025 Feb 17;56:44. doi: 10.1186/s13567-025-01454-7 (PMC11834572; doi:10.1186/s13567-025-01454-7)
Supplement: Supplementary file 1 — Additional file 1. Strains and plasmids involved in this study. The plasmids and strains involved in this study are listed in the table. [file 13567_2025_1454_MOESM1_ESM.docx]

**Additional file 1** **Strains and plasmids involved in this study**

| Strains or plasmids | Description | Reference or source |
| --- | --- | --- |
| Strains | | |
| WT | *R. anatipestifer* wild serotype 1, strong virulence | Laboratory collection |
| Δ*moxR* | *moxR* gene deletion strain, Spc^R^ | This study |
| CΔ*moxR* | *moxR* gene complemented strain, Spc^R^, Cfx^R^ | This study |
| WT::*moxR* | *moxR* gene overexpression strain, Spc^R^ | This study |
| WT::*moxRi* | *moxR* gene knockdown strain, Spc^R^ | This study |
| CΔ*moxR*::*moxRi* | *moxR* gene knockdown strain of CΔ*moxR*, Spc^R^, Cfx^R^ | This study |
| *E.coli* DH5α | Plasmid propagation strain | Weidi Co., Ltd. |
| *E.coli* BL21(DE3) | Protein overexpression strain | Weidi Co., Ltd. |
| *E.coli* X7213 | Diaminopimelic acid autotrophic strain used in transconjugation. λpir | Laboratory collection |
| *E.coli* DH5α λpir | Plasmid propagation strain. λpir | Weidi Co., Ltd. |
| Plasmids | | |
| pET28a | Protein overexpression strain | Laboratory collection |
| pET28a-MoxR | the plasmid used to express recombinant His_6_-MoxR | This study |
| pRE112 | Suicide vector, oriT, oriV, sacB, Cm^R^ | Laboratory collection |
| pRE112-*moxR*-LSR | pRE112 containing the fusion fragment  consisting of left and right arm of *moxR* and  spectinomycin resistance gene | This study |
| pRES-JX | shuttle vector, Cm^R^, Spc^R^ | Laboratory collection |
| pRES-JX-*moxR* | pRES-JX carrying *moxR* from RA-YM, Spc^R^ | This study |
| pRES-JX-*moxRi* | pRES-JX carrying *moxRi* from RA-YM, Spc^R^ | This study |
| pRES-*moxR*-Cfx | pRES-JX carrying *moxR* from RA-YM, Spc^R^, Cfx^R^ | This study |
| pRES-*moxR*-Cfx-*moxRi* | pRES-*moxR*-Cfx carrying *moxRi* from RA-YM, Spc^R^, Cfx^R^ | This study |
| pLMF03 | *B739_0921* promoter,  oriColE1, oripRA0726, Amp^R^, Cfx^R^ | [17] |
| pIC333 | source of spectinomycin resistance cassette | Laboratory collection |
