# Supplementary material for: MoxR effects as an ATPase on anti-stress and pathogenicity of Riemerella anatipestifer
Source: Vet Res. 2025 Feb 17;56:44. doi: 10.1186/s13567-025-01454-7 (PMC11834572; doi:10.1186/s13567-025-01454-7)
Supplement: Supplementary file 2 — Additional file 2. Primers used in this study. Primers for PCR and qPCR experiments were used in this study. [file 13567_2025_1454_MOESM2_ESM.docx]

**Additional file 2. Primers used in this study**

| Gene | Name | Sequence (5’-3’) | Reference or Designed |  |
| --- | --- | --- | --- | --- |
| Primers for constructing suicide plasmid | | |  | |
| *moxR*L | MoxRL-CZ-F | TTTGACTAgcatgcctatggaaaggaatatc | Designed |  |
|  | MoxRL-CZ-R | TTCGTTCCACTtcttccgcttgatgtaattctgc |  |  |
| *Spc* | MoxR-SPC-F | agcggaagaAGTGGAACGAAAACTCACGTTAAG | Designed |  |
|  | MoxR-SPC-R | CAGTAGTTTTAAAAGTAAGCACCTGTTATT |  |  |
| *moxR*R | MoxRR-CZ-F | GCTTACTTTTAAAACTACTGaactttattttatttgatacagaaaaatagatg | Designed |  |
|  | MoxRR-CZ-R | ccaagcttcttctagaggtacc |  |  |
| pRE112-GJ | JX-GJ-F | ggtacctctagaagaagctt | Designed |  |
|  | JX-GT-R | TCGTCGTCATCCTTGTAATCC |  |  |
| *mphes* | mphes-F | GGATTACAAGGATGACGACG | Designed |  |
|  | mphes-R | tccataggcatgcTAGTCAAAAGGATACCCATTAAAAAT |  |  |
| *16S rRNA* | 16S rRNA-F | AGAGTTTGATCCTGGCTCAGGATGA | Designed |  |
|  | 16S rRNA-R | ACGGCTACCTTGTTAGACTTAGCCCTAGTTACTA |  |  |
| *moxR* | MoxR-JD-F | gggcagtcttatatggtggatagatt | Designed |  |
|  | MoxR-JD-R | ccattctcattaccgctctttcgtcc |  |  |
| *Spc* | SPC-JD-F | TGGTGGTTTACGCATTAACAGCGAT | Designed |  |
|  | SPC-JD-R | TCGAGGTAATTTCACCAGTAGTCAC |  |  |
| Primers for constructing the complemented plasmid | | |  | |
| *Cfx* | CFX-F | agctccataaGTAATCTAAAAGCACTCCGAT | Designed |  |
|  | CFX-R | AATGAGACGTGAATTCGAGCTCGGTACC |  |  |
| *moxR* | pXY-*moxR*-F | GAGCTCGAATTCACGTCTCATTTTCGCCAAAAG | Designed |  |
|  | pXY-*moxR*-R | CTTTTAGATTACttatggagcttgaactctagc |  |  |
| pRES-JX | JX-*Nde*I-F | attctCATATGGTCCCAAACTGCA | Designed |  |
|  | JX-*spe*I-R | ACTAGTGATAGTATGACAGTCTAAGCTAGCA |  |  |
| pRES-JX-*moxR* | *moxR*-2-F | tgccatACTAGTttaaactttattaaaaatgta | Designed |  |
|  | *moxR*-2-R | ttagctttaGTCGACGATATATGAACATAAA |  |  |
| OmpA-anti2 | antiM-F | TTTTATGTTCATATATCGTCGACtaaagctaaagttttgattg | Designed |  |
|  | antiM-R | tacatttttaataaagtttaaACTAGTatggcagaattacatc |  |  |
| Primers for constructing the knockdown and overexpression plasmids | | |  | |
| *ompA* promoter | JX-ompA-F | ggACTAGTtaaagctaaagttttgattgcattagc | Designed |  |
|  | JX-ompA-R | ggCATATGagaattaaataattaaataattgataatgcaaa |  |  |
| Anti2/3 | Anti2-F | ccCATATGcattgtacctaccacatctgcag | Designed |  |
|  | Anti3-F | ccCATATGtcaatagggttttgcgttgcc |  |  |
|  | Anti23-R | GGGTACCatggcagaattacatcaagcgg |  |  |
| Anti4 | Anti4-F | ccCATATGataccacaggagttaccttaggaa | Designed |  |
|  | Anti4-R | GGGTACCatacagtttacgccagaccttc |  |  |
| Anti5 | Anti5-F | ccCATATGttatggagcttgaactctagcta | Designed |  |
|  | Anti5-R | gGGTACCggtgtctacatcagatattcctaag |  |  |
| *moxR* | JX-*moxR*-F | ccCATATGatggcagaattacatcaagcg | Designed |  |
|  | JX-*moxR*-R | cGGTACCttatggagcttgaactctagctaaa |  |  |
| Universal primers | JX-ompA-TY-F | ATCGTCGACGTAATAATGCCCGA | Designed |  |
|  | JX-ompA-TY-R | GTTTTGAATTGCTCTAACATACG |  |  |
| Primers for qPCR | | |  | |
| *recA* | recA-QP-F | GGTGTGTCTAAAGTTGGTGAGA | Designed |  |
|  | recA-QP-R | GTCCTAGCTTTGTGTCCTGATAA |  |  |
| *moxR* | *moxR*-Qp-F | GCAGATGTGGTAGGTACAATGA | Designed |  |
|  | *moxR*-Qp-R | TTGCTGGAGCACGGTTAAT |  |  |
| *DUF58* | DUF58-QP-F | atagatgcggcgcttatggg | Designed |  |
|  | DUF58-QP-R | gggttctttgaaacgagcagtt |  |  |
| *batD1* | *bat*D1-QP-F | agggcaggtacaaacaacgat | Designed |  |
|  | *bat*D1-QP-R | tccaagctctacctgcttgtt |  |  |
| *batA* | *batA*-QP-F | agaggcgttgatgagagtgc | Designed |  |
|  | *batA*-QP-R | acagctaccgccaaaccatc |  |  |
| *batB* | *bat*B-QP-F | cttcggtggtctctacgcaa | Designed |  |
|  | *bat*B-QP-R | tgcagcttcattgccttcgt |  |  |
| *batC* | *bat*C-QP-F | ggacagacgcctaaacctga | Designed |  |
|  | *bat*C-QP-R | gtgcagcatcacgctctttg |  |  |
| *batD2* | *bat*D2-QP-F | acggtgcgtttaaaaggggt | Designed |  |
|  | *bat*D2-QP-R | ccttcattccgtgctgggta |  |  |
| *moxR-DUF58* | MD-QP-F | agctagagttcaagctccataaa | Designed |  |
|  | MD-QP-R | ataagcgccgcatctatcc |  |  |
| Primers for constructing prokaryotic expression plasmids | | |  | |
| His-*moxR* | 28a-*moxR*-F | CGAATTCtttggagcttgaactctagctaaaactc | Designed |  |
|  | 28a-*moxR*-R | CGGATCCatggcagaattacatcaagcgg |  |  |
