# Supplementary material for: MoxR effects as an ATPase on anti-stress and pathogenicity of Riemerella anatipestifer
Source: Vet Res. 2025 Feb 17;56:44. doi: 10.1186/s13567-025-01454-7 (PMC11834572; doi:10.1186/s13567-025-01454-7)
Supplement: Supplementary file 3 — Additional file 3. ATP hydrolysis system with different concentrations of MoxR. The content of ATP was controlled to remain unchanged, and the ATP hydrolysis system was configured. After incubation, the content of phosphate groups in each system was determined by the molybdenum blue method. [file 13567_2025_1454_MOESM3_ESM.docx]

**Additional file 3** **ATP hydrolysis system 1**

| Components | Volume (μL) | | | | |
| --- | --- | --- | --- | --- | --- |
| Tris-HCl | 770 | 750 | 729 | 708 | 688 |
| MgCl_2_ | 100 | 100 | 100 | 100 | 100 |
| KCl | 100 | 100 | 100 | 100 | 100 |
| ATP | 30 | 30 | 30 | 30 | 30 |
| His_6_-MoxR | 0 | 20 | 41 | 62 | 82 |
