# Supplementary material for: MoxR effects as an ATPase on anti-stress and pathogenicity of Riemerella anatipestifer
Source: Vet Res. 2025 Feb 17;56:44. doi: 10.1186/s13567-025-01454-7 (PMC11834572; doi:10.1186/s13567-025-01454-7)
Supplement: Supplementary file 4 — Additional file 4. ATP hydrolysis system with different concentrations of ATP. The content of His6-MoxR was controlled to remain unchanged, and the ATP hydrolysis system was configured. After incubation, the content of phosphate groups in each system was determined by the molybdenum blue method. [file 13567_2025_1454_MOESM4_ESM.docx]

**Additional file 4 ATP hydrolysis system 2**

| Components | Volume (μL) | | | |
| --- | --- | --- | --- | --- |
| Tris-HCl | 780 | 730 | 710 | 688 |
| MgCl_2_ | 100 | 100 | 100 | 100 |
| KCl | 100 | 100 | 100 | 100 |
| His_6_-MoxR | 20 | 20 | 20 | 20 |
| ATP | 0 | 50 | 70 | 100 |
