# Supplementary material for: MoxR effects as an ATPase on anti-stress and pathogenicity of Riemerella anatipestifer
Source: Vet Res. 2025 Feb 17;56:44. doi: 10.1186/s13567-025-01454-7 (PMC11834572; doi:10.1186/s13567-025-01454-7)
Supplement: Supplementary file 7 — Additional file 7. Vaccination schedule. The dose inoculated in each group in the lethal dose of RA to ducklings. [file 13567_2025_1454_MOESM7_ESM.docx]

**Additional file 7. Vaccination schedule**

| Inoculation dose (CFU) | 10^4^ | 10^5^ | 10^6^ | 10^7^ | 10^8^ | 10^9^ |
| --- | --- | --- | --- | --- | --- | --- |
| RA-YM | 10 | 10 | 10 | 10 |  |  |
| Δ*moxR* |  | 10 | 10 | 10 | 10 | 10 |
| PBS |  | 10 | | | | |
