# Supplementary material for: MoxR effects as an ATPase on anti-stress and pathogenicity of Riemerella anatipestifer
Source: Vet Res. 2025 Feb 17;56:44. doi: 10.1186/s13567-025-01454-7 (PMC11834572; doi:10.1186/s13567-025-01454-7)
Supplement: Supplementary file 7 [file 13567_2025_1454_MOESM6_ESM.docx]

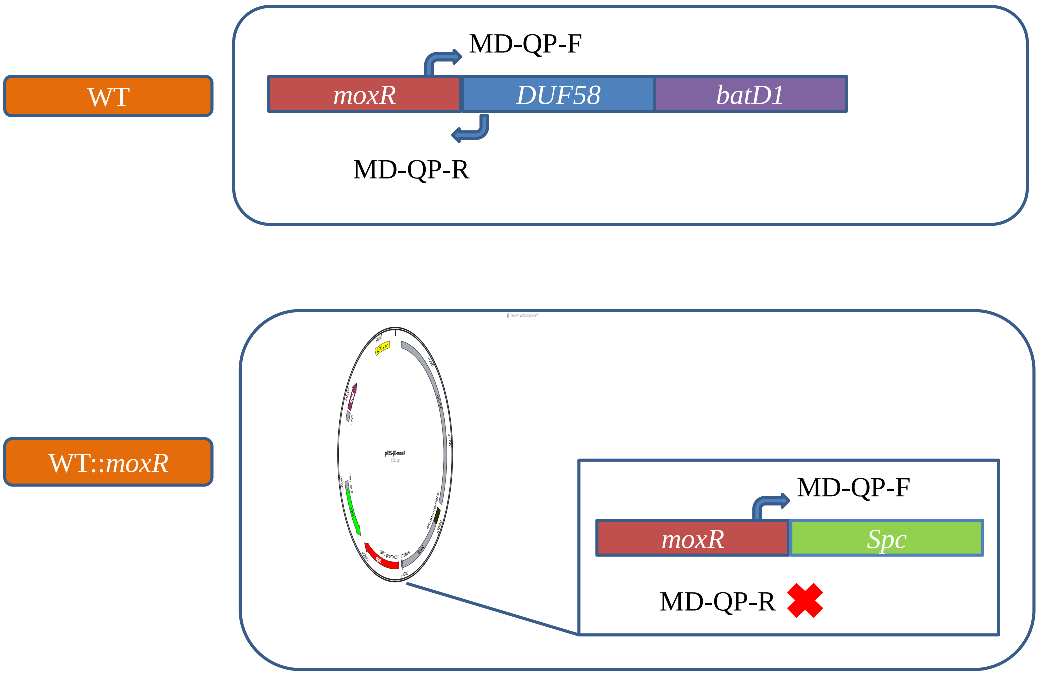
WT
MD-QP-F
moxR
DUF58
batD1
MD-QP-R
WT::moxR
MD-QP-F
moxR
Spc
MD-QP-R
