# Supplementary material for: MoxR effects as an ATPase on anti-stress and pathogenicity of Riemerella anatipestifer
Source: Vet Res. 2025 Feb 17;56:44. doi: 10.1186/s13567-025-01454-7 (PMC11834572; doi:10.1186/s13567-025-01454-7)
Supplement: Supplementary file 8 [file 13567_2025_1454_MOESM8_ESM.docx]

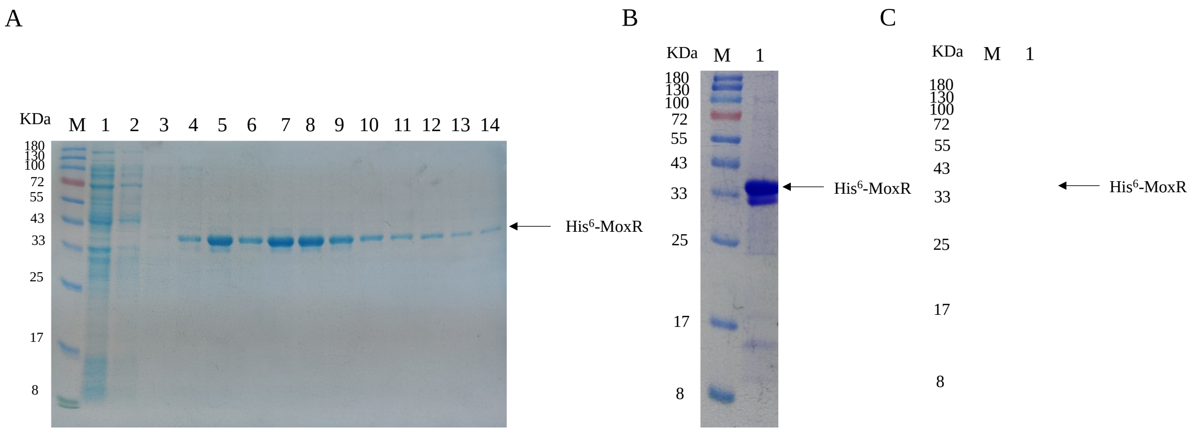

A
KDa
M 1 2 3 4 5 6 7 8 9 10 11 12 13 14
180
130
100
72
55
43
33
25
17
8
His6-MoxR
B
KDa
M 1
180
130
100
72
55
43
33
25
17
8
His6-MoxR
C
KDa
M 1
180
130
100
72
55
43
33
25
17
8
His6-MoxR
